# A molecular scheme for improved characterization of human embryonic stem cell lines

**DOI:** 10.1186/1741-7007-4-28

**Published:** 2006-08-18

**Authors:** Richard Josephson, Gregory Sykes, Ying Liu, Carol Ording, Weining Xu, Xianmin Zeng, Soojung Shin, Jeanne Loring, Anirban Maitra, Mahendra S Rao, Jonathan M Auerbach

**Affiliations:** 1 Stem Cell Center, American Type Culture Collection (ATCC^®^), Manassas, VA, USA; 2 Cell Biology Department, American Type Culture Collection (ATCC^®^), Manassas, VA, USA; 3 Laboratory of Neuroscience, National Institute on Aging, National Institutes of Health, Baltimore, MD, USA; 4 Buck Institute for Age Research, Novato, CA, USA; 5 Burnham Institute, La Jolla, CA, USA; 6 Departments of Pathology, Oncology, and Genetic Medicine, Johns Hopkins University School of Medicine, Baltimore, MD, USA; 7 Invitrogen Corporation, Carlsbad, CA, USA

## Abstract

**Background:**

Human embryonic stem cells (hESC) offer a renewable source of a wide range of cell types for use in research and cell-based therapies to treat disease. Inspection of protein markers provides important information about the current state of the cells and data for subsequent manipulations. However, hESC must be routinely analyzed at the genomic level to guard against deleterious changes during extensive propagation, expansion, and manipulation *in vitro*.

**Results:**

We found that short tandem repeat (STR) analysis, human leukocyte antigen (HLA) typing, single nucleotide polymorphism (SNP) genomic analysis, mitochondrial DNA sequencing, and gene expression analysis by microarray can be used to fully describe any hESC culture in terms of its identity, stability, and undifferentiated state.

**Conclusion:**

Here we describe, using molecular biology alone, a comprehensive characterization of 17 different hESC lines. The use of amplified nucleic acids means that for the first time full characterization of hESC lines can be performed with little time investment and a minimum of material. The information thus gained will facilitate comparison of lines and replication of results between laboratories.

## Background

Human embryonic stem cells (hESC) are a potentially limitless, albeit controversial, source of therapeutic cells for numerous diseases and injuries. It is likely that different hESC lines are best suited to different uses, but at present, it is rare for any laboratory to work with more than a few lines. One reason is the expense of these lines; another is that in the USA hESC are governed by a dual-track policy. Cell lines derived prior to 9 August 2001 (currently about 20 available lines) can be examined using federal funds, and currently much of the available information on hESC biology has been generated using these funds and cell lines [1]. However, cell lines derived after this date are far more numerous, but while it is legal to work on these lines using non-federal funds, information on their properties remains sparse. Government-funded researchers are reluctant to use these lines given the difficulties in accounting for federal and non-federal funds.

The lack of comparative analysis of hESC lines matters, because the properties and behavior of each line are uniquely shaped by their histories. It has become clear that different derivations produce hESC lines that are similar overall, but with inherent differences in gene expression, methylation status, X-chromosome inactivation, rate of self-renewal, and ability to differentiate [2-4]. More importantly, the behavior of cells and their phenotypic state changes as culture conditions and the stress to which they are subjected is altered, and permanent genomic changes frequently occur as passage numbers increase [5-7]. This has led to great difficulty in comparing results from one laboratory with another and even comparing results with different passages of the same cell line.

Therefore, thorough and routine characterization of hESC lines is essential to avoid compromising the validity of results. The most common characterization method for hESC is immunocytochemical analysis of a handful of markers, including SSEA-3, SSEA-4, TRA-1-60, TRA-1-80, and OCT-3/4 [8]. The next most frequent is reverse transcription PCR, which is used for those group of genes whose expression is involved in maintenance of the undifferentiated state [9,10]. While these assays certainly give indications of the undifferentiated state of the cells, they do not address other issues such as pluripotentiality or the degree of culture adaptation and genomic instability.

To facilitate comparisons among lines, the hESC research community has begun to develop a number of tools. Work is proceeding toward conditions that support the propagation of all lines [11], sets of markers that truly define the undifferentiated and unadapted state of the cells [7,12-14], and markers predictive of the differentiation capacity of the cells [15]. The work presented here is part of efforts to create a database of the properties of each line and to identify a reference standard for comparisons between laboratories. To this end, we have assembled a set of molecular tests for hESC lines that assess identity, stability of the nuclear and mitochondrial genomes, histocompatibility profile, and the undifferentiated state of the cells. Some of these assays have been previously performed on individual lines, but to our knowledge, no single group has used all of these tests on any single line, and few comparisons between lines have been made publicly available.

In this paper, we describe the analysis of multiple lines and show that this entire set of tests can be performed with a minimal sample size and over a short time period, and that these tests allow comparison of datasets across cell lines (a critical requirement to permit rapid progress in the field). We suggest that an internet database of hESC characterization data and standard reference materials will permit the research community to readily compare cell lines without the necessity of growing all lines in their own laboratory or tracking pre- and post-9 August cell lines.

## Results

The 17 cell lines analyzed are listed in Table 1. The lines were grown on feeders, or feeder-free on fibronectin, using bovine, human, or artificial serum according to the providers' protocols (see Methods).

### Measures of identity

**Figure 1:**
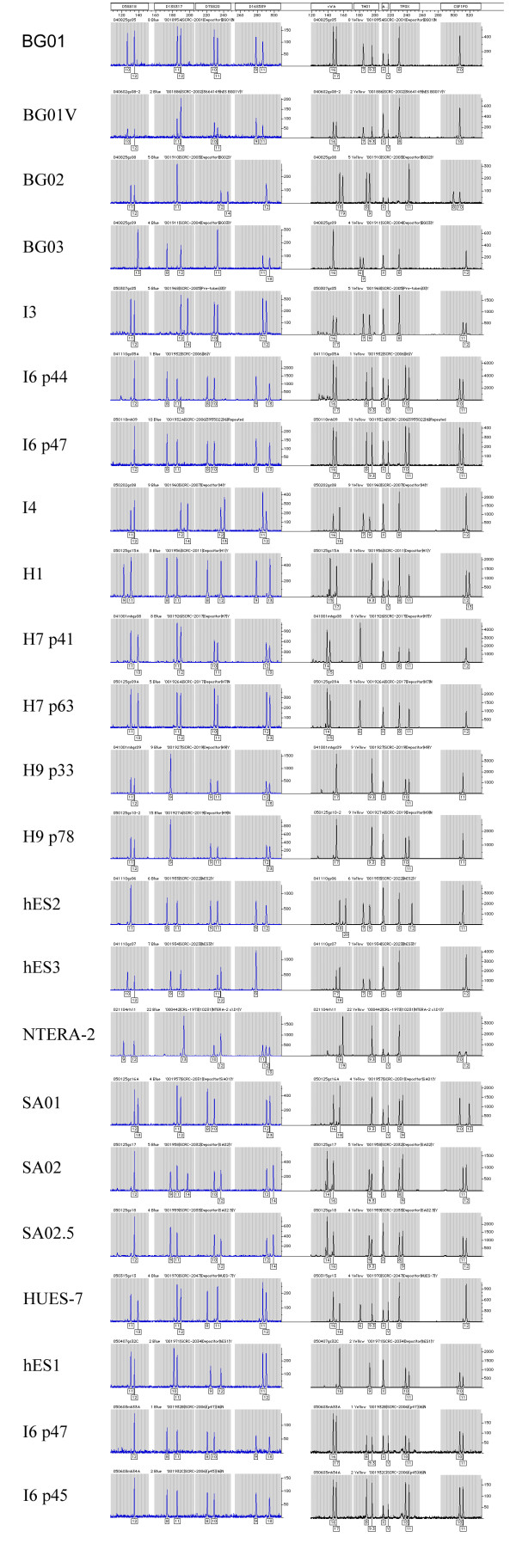
**STR analysis for identification**. STR profile of human embryonic stem cell lines. Loci analyzed by the PowerPlex 1.2 system (Promega) comprise D5S818, D13S317, D7S820, D16S539, vWA, TH01, TP0X, CSF1P0, and the sex-chromosome marker amelogenin. Data were collected on an ABI 310 genetic analyzer, and analyzed using Genescan 3.1 and Genotyper 2.0 software (all Applied Biosystems).

The results of short tandem repeat (STR) analysis of these lines are shown in Figure 1. The eight STR markers plus amelogenin used here are capable of identifying individual human genomes with a discrimination of 1 in hundreds of millions [16]. Each of the early passage lines studied here was shown to be genetically distinct from the others. Each late passage line or karyotypically abnormal subline (BG01V, SA02) is identical to the early passage line, showing no evidence of overgrowth by contaminating cells. Only two instances of possibly heterogeneous cultures are observed. Cultures of the I6 line were grown in two separate laboratories, and one of these cultures shows a reproducible abnormality in the chromosome 13 microsatellite marker D13S317. One culture at passage 44 shows a third allele (with 12 repeats) for this marker; the low intensity of the signal suggests either an abnormal subpopulation or lessened PCR efficiency. The second culture, grown independently to passage 47, shows two normal alleles at all loci. The second abnormal observation was also found at the D13S317 locus in the line SA02. This line shows three distinct alleles at passage 29, consistent with trisomy 13 as shown in the karyotype [17]. In contrast, the karyotypically normal subline SA02.5, which was subcloned from SA02, carries only two alleles on chromosome 13. The lines H7 and H9 show no changes visible by STR between lower and higher passage numbers.

The histocompatibility profile of each line was determined by sequence-specific oligonucleotide probes, and is summarized in Table 2. Each line has a unique histocompatibility profile, while the two sublines SA02.5 and BG01V are identical to their parental lines. Only the NTERA-2 embryonal carcinoma line shows an atypical histocompatibility profile, being homozygous at all tested loci.

### Measures of stability

Finer resolution analysis of genomic stability is afforded by single nucleotide polymorphism (SNP) assays. In this analysis, over 100,000 SNPs were assayed by microarray. The average SNP spacing in the genome is 26 kb.

**Figure 2:**
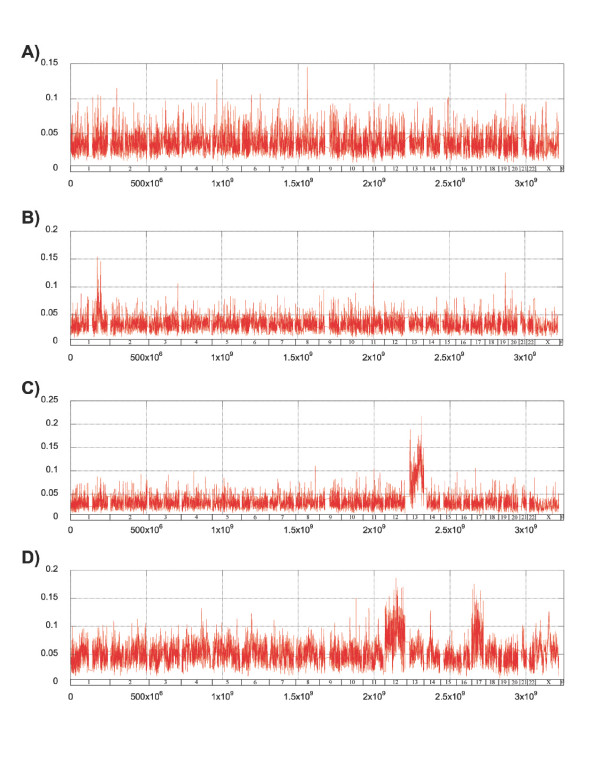
**Copy number analysis by SNP array**. DNA from hESC was amplified by PCR and hybridized to Sentrix BeadArrays. Paired lines (low versus high passage or karyotypic normal versus variant sublines) were compared at 101,000 loci. The 25-SNP moving average of the difference in allele intensity ratios is shown above the axis (red). **(A) **hESC line H7 compared at 41 and 63 passages. **(B) **hESC line H9 compared at 33 and 78 passages. **(C) **hESC line SA02.5 compared with the karyotypic variant SA02 (+13). **(D) **hESC line BG01 compared with the karyotypic variant subline BG01V (+12, +17, +X).

Figure 2 shows examples of hESC lines compared at low and high passage (H7 and H9), and comparison of karyotypically normal lines (BG01 and SA02.5) with karyotypically abnormal derivative lines (BG01V and SA02). SNP analysis can detect loss of heterozygosity (LOH) at any locus where two alleles were originally present, and is frequently used in analysis of tumors for discovery of potential oncogenes and anti-oncogenes [18]. In addition, comparison of intensity signals can be used to calculate copy number at all loci [19,20], and this has been recently used to detect genomic amplifications and deletions in hESC [6].

Comparison of the signal intensity and separation angle of all SNPs shows distinct discontinuities over certain chromosomes (Figure 2). This is the same approach used with Affymetrix SNP arrays to determine copy number [21]; however, reference BeadArray data from known diploid loci is required to translate intensities into copy number. Nevertheless, local changes in the allele intensity ratio (theta) are seen in chromosomes 12, 17, and a portion of chromosome X of BG01V, chromosome 13 of SA02, and within the q arm of chromosome 1 in H9. All of these areas have previously been shown to be duplicated in these cell lines [6]. No other significant discontinuities were found for any other chromosome of any line.

Sequencing of the entire mitochondrial genome (16,544bp) was performed using an oligonucleotide sequencing microarray (Affymetrix MitoChip v2.0), as previously described [6]. Briefly, 'early' and 'late' passages of nine paired hESC lines (BG01, BG02, BG03, HES2, HES3, SA01, SA02, H7, and H9) were sequenced for alterations in mtDNA in the course of *in vitro *passage. The results of this study have been published elsewhere [6], and six heteroplasmic sequence alterations occurring in two of nine (22%) later passage hESC lines were identified and confirmed by conventional dideoxy sequencing. The raw MitoChip data on the nine hESC lines is available on request (from AM).

### Measures of pluripotency

**Figure 3:**
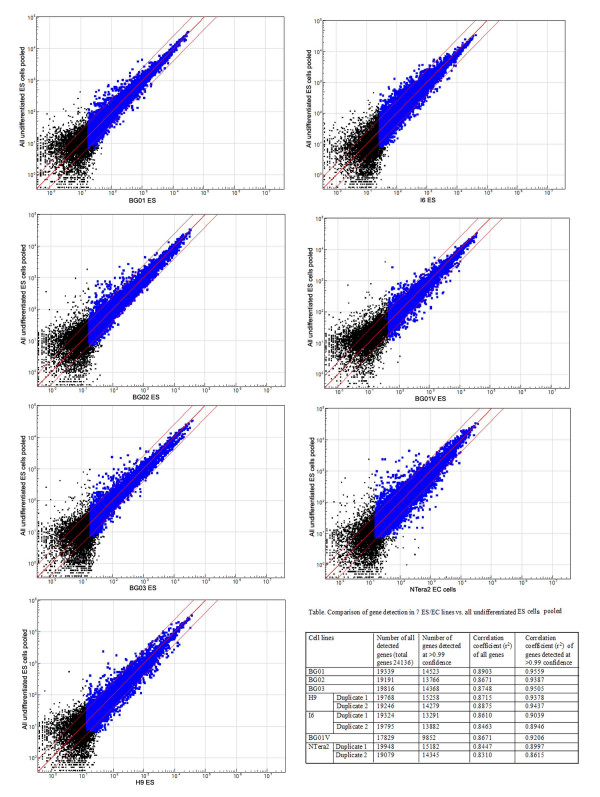
**Gene expression analysis by BeadArray**. RNA samples from six human ES cell lines and one human EC line were analyzed by Illumina BeadArray, and scatterplots were made by comparing individual lines with pooled ES cell samples. Genes that were detected at >0.99 confidence from the array were labeled blue, and genes falling outside of the red lines were detected at >2.5-fold difference. Total gene numbers and values of the correlation coefficient (*r*^2^) of all comparisons were summarized in the table.

Gene-expression analysis was performed by BeadArray (Illumina) to assay the transcriptome of each line. RNA expression profiles of several lines were directly compared with RNA from a pool of three undifferentiated lines previously used to compare expression by EST enumeration and MPSS [15,22] (Figure 3). The normal lines tested (H9, I6, BG01, BG02, and BG03) show an average correlation coefficient (*r*^2^) of 0.93 for genes detected with high confidence (>0.99). Karyotypically abnormal ES and EC lines (BG01V and NTERA-2) produce *r*^2 ^values of 0.92 and 0.88, respectively. This suggests that overall gene expression is more sensitive to the difference between normal and culture-adapted lines than is the use of immunocytochemical markers.

Not all genes contribute equally to the pluripotency of hESC, of course. Recent work comparing the transcriptomes of multiple hESC lines has identified core sets of 105 ES-specific genes [12] and 194 genes specifically upregulated in differentiating embryoid bodies [14], which have been used to create focused arrays for hESC testing. Of the ES-specific genes, 96 could be identified and detected at high confidence in our pooled hESC lines (Table 4). Nearly all of these were detected at similar levels in all of the individual hESC lines tested (H9, BG01, BG01V, BG02, BG03 and I6) and in the EC line NTERA-2. Every line but BG01 under- or over-expressed by 2.5-fold at least one ES marker, especially I6, which underexpressed 11 genes including *TDGF-1*, *REX-1*, and *UTF-1*. Few ES-specific genes were overexpressed, except for the variant line BG01V, which overexpressed four genes, including *ABCG2*, and NTERA-2, which overexpressed two genes, including the germ cell tumor marker *GDF3*.

Of the 192 differentiation-specific genes, 168 could be detected at a significant level in the pooled hESC lines. Approximately 89–96% of these were also detected in any individual line, except for BG01V, in which 134 genes were significantly expressed. This agrees with previous findings of low-level expression of diverse differentiation markers in undifferentiated hESC [23]. NTERA-2 and I6 underexpressed the greatest number of these markers, while H9 and NTERA-2 also overexpressed six EB-specific genes each. In H9, three of these were collagen genes, suggesting the presence of human fibroblasts possibly differentiated from the hESC grown under feeder-free conditions.

## Discussion

The technical demands of culturing human embryonic stem cells are already well known in the field. It is now becoming clearer that frequent characterization of the cells in culture must be added to the burden. The present data confirm the general finding that hESC lines in culture accumulate genomic, epigenetic, and mitochondrial changes within as few as 22 passages [6]. As a result, laboratories attempting to expand hESC lines must subject them to frequent characterization tests sensitive to aberrations below the resolution of G-banded karyotypes. The number and frequency of required tests also increases the value of rapid and convenient test methods, such as the many array-based molecular tests currently on the market or in development. This manuscript presents a molecular characterization scheme for hESC lines that helps to define the state of the cells. The tests include measures of identity, stability, and self-renewal, and the undifferentiated state of the cells. In addition, we have provided data on potential reference standards for hESC research. We show by examples that each of these tests offers important insights into the state of the hESC line and that the combination of these tests provides a unique characterization profile of a given cell line that is sensitive to the deleterious changes that can occur in culture.

This initiative, led by the American Type Culture Collection (ATCC) and supported by most of the providers of hESC lines, represents a complementary effort that uses cell lines approved by the National Institutes of Health to develop a basis for comparison across different laboratories. Equally important, by providing data in the same format on a new post-9 August line, it facilitates the direct comparison of lines eligible and ineligible for federal funding to determine which cell line is best suited to the individual researcher's needs. Such a comparison has not to our knowledge been previously performed, as it is largely impossible for most researchers in the USA.

Our estimate of testing costs for this characterization scheme is that these can be completed for a single line for approximately $2500. The identity, stability, and undifferentiated state of lines can be assessed with less than 5 μg of genomic DNA and 100 ng of total RNA. This can be obtained from 2–3 million cells, comparable with the amount required to run a single karyotype analysis using Giemsa-banded metaphase spreads. We further note that it will not be necessary for every researcher to repeatedly perform every one of these tests. We have identified commercial service providers both within and outside the USA, which can perform these analyses, and ATCC and other banks have offered to similarly characterize the lines they maintain.

The characterization scheme is composed of tests chosen on the basis of comprehensiveness, wide availability, and cost efficiency. For example, STR analysis as a measure of identity is a simple PCR-based analysis that is available in a kit from multiple providers, and can be readily run in any laboratory. One advantage of this assay is that it also readily distinguishes male and female samples, and in theory can be used to examine relatedness between samples and the larger database sets that have been generated for other purposes. ATCC is developing a comprehensive database of DNA profiles of hESC and other human cell lines based on STR loci [24]. This test allows for discrimination of at least 1 in 100 million individuals [16].

HLA typing of hESC lines is essential, both for future use in transplantation and for the immediate goal of estimating how many hESC lines are required to meet the needs of all potential patients [25]. HLA typing at the allelic level included HLA-A, HLA-B, HLA-C, HLA-DRB, and HLA-DQB loci.

SNP arrays are commonly used for mapping new markers of genetic disease and for detecting LOH in cancer. Recently, algorithms have been developed that allow oligonucleotide SNP arrays to measure chromosomal copy number at high resolution [19,20]. This expands the utility of SNPs to detect non-reciprocal translocations, aneuploidy, and partial amplifications or deletions of chromosomes. Maitra et al have used this method to show that hESC in culture can acquire amplifications or deletions of small chromosomal regions [6]. The SNP array method is of great value in characterizing hESC lines, as it improves the resolution for detecting genetic alterations. Conventional Giemsa-stained karyotypes or spectral karyotypes have a resolution of about 1–5 Mb. Comparative genomic hybridization (CGH) of metaphase spreads has a resolution of approximately 20 Mb, and array-based CGH with genomic or cDNA clones has improved this to <1 Mb. Bacterial artificial chromosome clone arrays have now improved the resolution to <100 kb. In contrast, currently available arrays of 100,000 SNPs have a potential resolution of about 25 kb. With over 2 million SNPs already mapped in the human genome, increased density arrays could drop this even further.

Like SNP genotyping, sequencing of the mitochondrial DNA offers the dual benefits of measuring identity (across 16,544 bases comprising a large number of polymorphic haplotypes) as well as stability over passages. Mitochondrial genes accumulate mutations in the somatic cells of aging animals. Mice in which mtDNA damage is accelerated show numerous symptoms of premature aging [26]. The connections between tissue stem cells and aging are still controversial, but it has been demonstrated that changes in mitochondrial properties correlate with reduced competence of adult stem cells [27]. Thus, although mtDNA mutations are quite common in somatic cells, the functional consequences may be greater in stem cells. Array-based sequencing of mtDNA is cost effective, and the reduced number of reactions needed in long-range PCR reactions and the automated analysis of genotype data using the RA Tools software (see Methods) significantly decreases analysis time.

Measures of identity and stability are generally of less concern to researchers growing hESCs than are the potency and undifferentiated state of the cells. These qualities are generally assessed by antibody staining for protein biomarkers. However, with the ability to rapidly assess expression of all human genes, it is possible to measure the expression of undifferentiated markers and any conceivable differentiation marker in a single step. The gene-expression profile of pluripotent hESCs has not been completely defined, but much work has been carried out toward defining common sets in undifferentiated and differentiating hESCs [12-14]. The use of this microarray method to distinguish undifferentiated and differentiated hESC cultures has been demonstrated [27]. This gives the possibility of assigning a quality score to any hESC culture, which will be of value in improving culture methods.

The main drawback of these molecular methods is that the cells are assayed as an amalgam, so the ability to detect differentiated cells or aberrant subpopulations must be demonstrated. For example, we have estimated that at least 20% of the population must carry a genetic abnormality before it can be detected by a SNP array (data not shown). Currently, this method is often used to screen for LOH in cancer. Normal cells within the tumor severely degrade the sensitivity of LOH detection when as few as 10% are present, but copy-number detection is less sensitive to mixed populations [28]. Similarly, the presence of low levels of differentiated cells is likely to escape notice when surveying global gene expression. The data presented here shows the overexpression of collagen genes in a sample of hESC cultured in feeder-free conditions, which suggests that the microarray method can detect differentiating subpopulations, but there is no way to retrospectively determine the level of differentiation.

Owing to the demands of culturing hESCs, it is rare that any one laboratory will bring together numerous lines to compare them under identical conditions. Thus, comparisons must be facilitated between laboratories by the promulgation of standard reference materials. The present work has thus compared multiple hESC lines to several potential reference materials, each having advantages in different areas of hESC research.

The greatest need for standards is in the area of gene-expression studies, as whole genome arrays are still expensive enough to discourage broad comparisons in any one laboratory, and the results are not easily compared between laboratories. For gene expression studies, we have created a pool of RNA from three independent hESC lines. This pool shows great concordance of expression with individual normal hESC lines, as the correlation coefficients (*r*^2^) with normal lines average 0.87, or 0.93 for genes expressed at high confidence levels. Even if the line H9, one of the lines in the pooled samples, is excluded, the *r*^2 ^of highly confident expression for normal ES lines averages 0.93. Thus, the pooled sample shows high colinearity with hESC lines of diverse origin. As the RNA pool can be captured in the form of a cDNA library relatively inexpensively, it can easily be widely disseminated.

Alternatively, we have also provided data on embryonal carcinoma and karyotypically abnormal hESC lines, which are potential reference materials. As these lines are freely available, relatively inexpensive, and not subject to licensing and patent permissions, it is feasible to run these lines as internal/normalization controls. Internal reference standards also allow normalization of data to posted datasets available worldwide. NTERA-2 is an embryonal carcinoma line that expresses the same early embryo antigens now used to identify undifferentiated hESC. In global gene-expression studies, such as the data presented here, NTERA-2 closely mimics hESC lines, showing an average *r*^2 ^with the hESC pool of 0.84 for all genes and 0.88 for genes at high confidence. Reference material in the form of RNA or cDNA could easily be produced in large lots from this cell line, for distribution around the world. The NTERA-2 line also has advantages for antibody-based comparisons, as the line is well adapted to culture and grows quickly without feeder layers, attachment matrices, or the addition of any specialized growth factors other than serum. If the phenotype of NTERA-2 were compared between laboratories, this last would probably be the major source of variation. Thus, an effort could be made to introduce standardized serum-free conditions for the growth of NTERA-2 and validate the reproducibility of its gene expression across laboratories in order to create an unlimited source of reference material for hESC.

While the undifferentiated phenotype of NTERA-2 is quite similar to that of hESCs, its behavior in differentiation paradigms is quite different. It shows little spontaneous differentiation in culture without induction by retinoic acid or other chemicals [29,30]. Therefore, it may be more advantageous to have a reference material that is grown in conditions identical to normal hESC and shows a similar capacity for differentiation *in vitro *[31] and *in vivo *[32]. The aneuploid line BG01V has these characteristics. Compared with NTERA-2, the overall gene-expression profile of BG01V is more similar to undifferentiated hESC lines (*r*^2 ^= 0.87 for all genes, 0.92 for genes at high confidence). The gene expression of differentiating cultures is being examined, but is much more likely to mimic differentiating hESCs than NTERA-2 would. For whole-cell studies of differentiation, BG01V is available at low cost. The growth conditions for BG01V, as for hESC in general, have not yet been standardized to ensure reproducible results across laboratories.

## Conclusion

In summary, we have found that the molecular characterization of hESC can be performed with minimal material, time, and expense. The series of tests presented here provides a method for frequent monitoring of many of the properties thought to be involved in stem-cell pluripotency. We believe that such characterization provides a useful set of results that will aid the cooperative international stem-cell effort, and will allow progress to be more rapid than in the past. We also hope that as cooperative standards become more widely accepted, commercial organizations will see fit to offer these as tools or kits to make the work of individual researchers easier.

## Methods

### Establishment and culture of human embryonic stem cell lines

All hESC lines were grown under similar conditions, with variations dictated by the providers' protocols. The standard culture medium was Dulbecco minimal essential medium (DMEM)/F12-Glutamax, 20% KnockOut Serum Replacement (KSR), 2 mM nonessential amino acids, 100 μM beta-mercaptoethanol (all from Invitrogen, Carlsbad, CA, USA), 50 μg/ml penicillin/streptomycin, and 4 ng/ml human recombinant basic fibroblast growth factor (FGF2; PeproTech Inc., Rocky Hill, NJ, USA) Feeder-free cultures were supplemented with FGF2 at 20 ng/ml.

TE06 (I6): Cells were cultured for 30 passages on mouse embryonic fibroblast (MEF) layers in KnockOut DMEM supplemented as above, except for the use of 5% fetal calf serum and 15% KSR (Invitrogen).

BG01, BG02, BG03 and BG01V: Cells were maintained under feeder-free condition on fibronectin-coated plates in medium that had been conditioned by mouse-embryo fibroblasts for 24 hours.

WA09 (H9): Human ES cell line H9 (WiCell, Madison, WI, USA) were cultured on feeder layers derived from mitotically inactivated WA09-derived mesenchyme or under feeder-free conditions on Matrigel- or human laminin-coated plates for at least 10 passages.

HUES-7: cells were maintained on a feeder layer of mitomycin C-treated CF-1 mouse embryonic fibroblasts (MEF) (ATCC, SCRC-1040.2). Growth medium was ES-DMEM (ATCC) supplemented with plasmanate (10%) (Bayer), KSR (10%; Invitrogen), L-alanyl-L-glutamine (2.0 mM; ATCC), non-essential amino acids (1X; ATCC), β-mercaptoethanol (0.055 mM; Invitrogen), penicillin (100 IU/mL) and streptomycin (100 μg/mL) (both ATCC), and FGF2 (12 ng/mL; R&D Systems, Inc.).

Cells were passaged every 4–5 days using collagenase IV (200 U/mL; Invitrogen) except HUES-7, which were passaged using 0.05% trypsin/EDTA (Invitrogen).

### STR typing

Frozen cell pellets were resuspended in 1× phosphate-buffered saline (PBS). A 20-mL aliquot was spotted onto a labeled FTA^® ^card (Whatman) and allowed to dry. The FTA card lyses the cells on contact and binds the DNA to the paper surface. Prior to PCR, a portion of the dried spot was removed with a Harris punch, washed three times with purification reagent (Whatman), washed once with Tris-EDTA (TE) buffer (pH 8.0), and allowed to dry. STR analysis was conducted using the multiplex-PCR-based PowerPlex 1.2 kit (Promega Corporation). Loci analyzed include D5S818, D13S317, D7S820, D16S539, vWA, TH01, amelogenin, TP0X and CSF1P0. Electropherogram data were collected on an ABI 310 genetic analyzer (Applied Biosystems). Data was analyzed using Genescan 3.1 and Genotyper 2.0 software (Applied Biosystems). The resulting profile was imported into an in-house database and screened against all other baseline profiles of all samples tested by the ATCC. STR analysis of the NTERA-2 cell line was performed similarly using isolated genomic DNAs.

### HLA typing

Genomic DNA was isolated from cells using the GenElute™ mammalian genomic DNA miniprep kit (Sigma-Aldrich, Inc.). HLA DNA typing was performed by utilizing hybridization of PCR-amplified DNA with sequence-specific oligonucleotide probes (SSOP) (Tepnel Lifecodes Corporation). The target DNA is amplified by PCR and then allowed to denature and rehybridize to complementary DNA probes conjugated to fluorescently coded microspheres. A flow analyzer identifies the fluorescent intensity on each microsphere, and the determined HLA type is based on the reaction pattern compared with patterns associated with public HLA gene sequences. Assays were performed to determine the HLA-A, HLA-B, HLA-C, HLA-DRB, and HLA-DQB loci.

### SNP analysis

Genomic DNA isolated as above was amplified following the Infinium™ whole genome genotyping assay [32] and hybridized to a prototype Sentrix BeadArray (Illumina Inc., San Diego, CA, USA). Allele calls were made by GenCall software (Illumina Inc.).

### Gene expression analysis

Feeder-free cultures were prepared for gene expression analysis by manually harvesting individual colonies with uniform typical undifferentiated ES cell morphology. Feeder-containing cultures were harvested by scraping both ES and feeder cells. WA01 (H1), WA07 (H2), and WA09 (H9) cells were cultured under feeder-free conditions, and pooled prior to RNA isolation.

RNA was isolated from cultured cells using the Qiagen RNEasy kit (Qiagen, Inc, Valencia, CA, USA). Sample amplification was performed using 100 ng of total RNA as input material by the method of Van Gelder et al [33] using the Illumina^® ^RNA amplification kit (Ambion Inc., Austin, TX, USA) following the manufacturer's instructions. Labeling was achieved by incorporating biotin-16-UTP (Perkin-Elmer Life and Analytical Sciences, Boston, MA, USA) present at a ratio of 1:1 with unlabeled UTP. Labeled, amplified material (700 ng per array) was hybridized to a pilot version of the Illumina Ref-8 BeadChip according to the manufacturer's instructions (Illumina Inc.). Arrays were washed then stained with Amersham FluoroLink streptavidin-Cy3 (GE Healthcare Bio-Sciences, Little Chalfont, Buckinghamshire, UK) following the BeadChip manual. Arrays were scanned with an Illumina BeadArray Reader confocal scanner according to the manufacturer's instructions. Array data processing and analysis was performed using Illumina BeadStudio software.

### Mitochondrial DNA sequencing

Genomic DNA was extracted from paired hESC lines, and PCR amplified in three long-range PCR reactions as previously described [34]. Amplified DNA was pooled, fragmented. and labeled as described in the Affymetrix CustomSeq™ resequencing protocol. MitoChips were hybridized overnight, washed on the Affymetrix fluidics station using the pre-programmed CustomSeq™ resequencing wash protocols, and scanned. Data analysis was performed using RA Tools, a modified version of the previously described adaptive background genotype-calling scheme (ABACUS) [35]; the open-source software is available from the *Drosophila *Population Genomics Project [36]. Briefly, RA Tools uses an objective statistical framework to assign each genotype call a 'quality score', which is the difference between the log_10 _likelihood of the best-fitting and the second-best-fitting statistical model for assigning a genotype at any position on the sequencing array. The total quality-score threshold (totThresh) is the quality score that a given base has to exceed in order to be called. Increasing this value requires increased support for base calls, and consequently, fewer bases are called. Bases that fail to reach this threshold are called 'N.' The optimum total threshold quality score is determined empirically to be 12, which yields the highest base-call rate with the lowest discrepancy between genotypes for replicate samples. Confirmation of array-based sequencing was performed with a dye-terminator platform using the ABI Big Dye cycle sequencing kit (Applied Biosystems).

## Authors' contributions

GS performed the STR analysis of all lines. YL, SS, and JL performed gene expression analysis. CO, WX, and XZ grew hESC lines for analysis and provided materials to outside testers. RJ analyzed the genotyping data. AM developed and performed the mitochondrial-DNA sequencing. MSR and JMA developed the characterization scheme and coordinated all materials and tests. RJ and MSR wrote the manuscript.

## Figures and Tables

**Table 1:** Cell lines analyzed

Embryonic stem cell line	NIH Registry name	Passage no	Reference	STR	HLA	SNP	mtDNA	Gene exp.
BG01	BG01	P43	Brimble *et al*, 2004	+	+	+	+	+
BG01V	BG01V	P25	Zeng *et al*, 2004	+	+	+	+	+
BG02	BG02	P13, P14	Brimble *et al*, 2004	+	+		+	+
BG03	BG03	P14	Brimble *et al*, 2004	+	+		+	+
I3	TE03	P38	Amit and Itskovitz-Eldor, 2002	+				
I6	TE06	P44, P47	Amit and Itskovitz-Eldor 2002	+	+			+
I4	TE04	P29	Amit and Itskovitz-Eldor 2002	+	+			
H1	WA01	P80	Thomson *et al*, 1998	+	+		+	
H7	WA07	P41, P63	Thomson *et al*, 1998	+	+	+	+	
H9	WA09	P33, P78	Thomson *et al*, 1998	+	+	+	+	+
hES2	ES02	P55, P146, P147	Reubinoff *et al*, 2000	+	+		+	
hES3	ES03	P59, P92, P93	Reubinoff *et al*, 2000	+	+		+	
SA01	SA01	P14, P25, P32, P60	Heins *et al*, 2004	+	+		+	
SA02	SA02	P29	Heins *et al*, 2004	+	+	+	+	
SA02.5	SA02.5	P155+49		+	+	+	+	
Relicell™ hES1	Not registered	P30	Mandal *et al*, 2006	+	+			
HUES-7	Not registered	P14	Cowan *et al*, 2004	+	+			

+ indicates assays performed and reported in this publication.

Exp., expression.

**Table 2:** STR results

	Locus ID								
	
Designation	D5S818	D13S317	D7S820	D16S539	vWA	TH01	Amelogenin	TPOX	CSF1PO
BG01	10, 12	11, 12	10, 11	9, 11	16, 17	7, 9.3	X, Y	8	10
BG01V	10, 12	11, 12	10, 11	9, 11	16, 17	7, 9.3	X, Y	8	10
BG02	11, 12	11	12, 14	12	18, 19	8, 9	X, Y	11	8, 10
BG03	13	8, 12	11	11, 13	16	6, 7	X	8	12
I3	11, 12	12, 14	10, 11	11, 12	16, 17	7, 9	X	8	11, 12
I6-P44	12	8, 11, 12	8, 10	9, 13	16, 17	8, 9.3	X, Y	10, 11	10, 11
I6-P47	12	8, 11	8, 10	9, 13	16, 17	8, 9.3	X, Y	10, 11	10, 11
I4	11, 12	12, 14	12, 13	11, 12	16, 18	7, 9	X	8	12
H1	9, 11	8, 11	8, 12	9, 13	15, 17	9.3	X, Y	8, 11	12, 13
H7-P41	11, 13	11, 12	10, 11	12, 13	14, 15	6	X	8, 11	12
H7-P63	11, 13	11, 12	10, 11	12, 13	14, 15	6	X	8, 11	12
H9-P33	11, 12	9	9, 11	12, 13	17	9.3	X	10, 11	11
H9-P78	11, 12	9	9, 11	12, 13	17	9.3	X	10, 11	11
hES2	11	8, 11	9, 11	9, 12	18, 20	7, 9	X	8, 12	11
hES3	10, 12	9, 12	11, 12	9	17, 18	7, 9	X	8	12
SA01	12, 13	11, 12	8, 10	12, 13	16, 18	9.3	X, Y	8, 9	10, 13
SA02	12	9, 11, 14	10, 12	12, 14	14, 16	9, 9.3	X	8, 9	11, 12
SA02.5	12	9, 11	10, 12	12, 14	14, 16	9, 9.3	X	8, 9	11, 12
Relicell™ hES1	11, 12	10, 11	9, 12	11, 12	18	9	X	10, 11	10, 11
HUES-7	11, 13	11, 12	8, 11	11, 12	16, 18	6, 9.3	X, Y	8	12
NTERA-2	9, 12	13	10, 12	11,12,13	18, 19	9.3	X, Y	8	10, 12

**Table 3:** HLA profiles

Locus		HLA-A	HLA-B	HLA-C	DRB1	DRB3	DRB5	DQB1
BG01	Allele code	02XX, 03XX	35XX, 49XX	04XX, 07XX	03VEW, 04DAZ	01ANEX		02XX, 03XX
	**Serological equivalent**	**A2, A3**	**B35, B49**	**Cw4, Cw7**	**DR3, DR4**	**DR52**		**DQ2, DQ3**
BG01V	Allele code	02XX, 03XX	35XX, 49XX	04XX, 07XX	03VEW, 04DAZ	01ANEX		02XX, 03XX
	**Serological equivalent**	**A2, A3**	**B35, B49**	**Cw4, Cw7**	**DR3, DR4**	**DR52**		**DQ2, DQ3**
BG02	Allele code	24AZMA, 68AZMC	3511, 35MWA	03XX, 04XX	15ANVF, 1602		01KBE	03NX, 0601
	**Serological equivalent**	**A24, A68**	**B35, B35**	**Cw3, Cw4**	**DR15, DR16**		**DR51**	**DQ3, DQ6**
BG03	Allele code	24AZMA, 68AZMC	44AZZX, 44AZNT	05XX	04ANUK, 04JRG			03XX
	**Serological equivalen**t	**A24, A68**	**B44, B44**	**Cw5**	**DR4, DR4**			**DQ3**
I6	Allele code	01ADJA, 26TJW	38AF, 44AZKJ	04XX, 12XX	04BK, 07YRE			02XX, 03XX
	**Serological equivalent**	**A1, A26**	**B38, B44**	**Cw4**, none	**DR4, DR7**			**DQ2, DQ3**
I4	Allele code	03ANPZ, 24BCKM	3502, 38AF	04XX, 12XX	13ANYX, 13AMGH	01ANUB		06XX
	**Serological equivalent**	**A3, A24**	**B35, B38**	**Cw4**, none	**DR13, DR13**	**DR52**		**DQ6**
H1	Allele code	02BBNK, 03ANPZ	08ARGR, 35ASKY	04XX, 07XX	01AMFN, 03VEW	01ANEX		02XX, 05XX
	**Serological equivalent**	**A2, A3**	**B8, B35**	**Cw4, Cw7**	**DR1, DR3**	**DR52**		**DQ2, DQ5**
H7	Allele code	02BABR, 25AC	18AZYW, 35AZZE	04XX, 12XX	07SAF, 11BBBW	02ARZT		02XX, 03XX
	**Serological equivalent**	**A2, A25**	**B18, B35**	**Cw4**, none	**DR7, DR11**	**DR52**		**DQ2, DQ3**
H9	Allele code	02AZKW, 03ARDP	35JXF, 44AZZW	04XX, 07XX	15BCFX, 1601		01AUMM	05XX, 06XX
	**Serological equivalent**	**A2, A3**	**B35, B44**	**Cw4, Cw7**	**D15, D16**		**DR51**	**DQ5, DQ6**
hES-2	Allele code	0203, 02BBFJ	46AB, 48AC	01XX, 08XX	04ASVM,15AUAM		01REZ	04XX, 06XX
	**Serological equivalent**	**A2, A2**	**B46, B48**	**Cw1, Cw8**	**DR4, DR15**		**DR51**	**DQ4, DQ6**
hES-3	Allele code	11ZPJ, 33DWH	40ADGG, 55GEJ	01XX, 07XX	12ARRT, 12ATDH	01AUPH		06XX
	**Serological equivalent**	**A11, A33**	**B60, B55**	**Cw1, Cw7**	**DR12, DR12**	**DR52**		**DQ6**
SA01	Allele code	01ADVC, 02AZUK	07AZMH, 35RYF	04XX, 07XX	13ASTS, 15UVF	02UPA	01REZ	06XX
	**Serological equivalent**	**A1, A2**	**B7, B35**	**Cw4, Cw7**	**DR13, DR15**	**DR52**	**DR51**	**DQ6**
SA02	Allele code	01ADVC, 23ARDZ	07AZHW, 51AZJX	07XX, 15XX	14ASYK, 14AMRN	02AJHF		05XX
	**Serological equivalent**	**A1, A23**	**B7, B51**	**Cw7**, none	**DR14, DR14**	**DR52**		**DQ5**
SA02.5	Allele code	01ADVC, 23ARDZ	07AZHW, 51AZJX	07XX, 15XX	14ASYK, 14AMRN	02AJHF		05XX
	**Serological equivalent**	**A1, A23**	**B7, B51**	**Cw7**, none	**DR14, DR14**	**DR52**		**DQ5**
Relicell hES1	Allele code	01WUS, 02BCKD	5601, 35ASKY	01XX, 04XX	01AMFN, 01CRM			05XX
	**Serological equivalent**	**A1, A2**	**B56, B35**	**Cw1, Cw4**	**DR1, DR1**			**DQ5**
HUES7	Allele code	02BBNK, 03ANPZ	35CU, 51ASMM	04XX, 14XX	04YEP, 15AK		01XTV	03XX, 06XX
	**Serological equivalent**	**A2, A3**	**B35, B51**	**Cw4**, none	**DR4, DR15**		**DR51**	**DQ3, DQ6**
NTERA-2	Allele code	01ADJA	08AZMJ	07XX	03ASDU	01ASFC		02XX
	**Serological equivalent**	**A1**	**B8**	**Cw7**	**DR3**	**DR52**		**DQ2**

**Table 4:** Expression of hESC- and EB- specific genes

	hESC pool	H9	NTERA-2	BG01	BG02	BG01V	BG03	I6
Undifferentiated hESC genes (*n *= 104)	96	95	94	92	94	88	94	92
Underexpressed <0.4 ×	-	2	4	0	1	1	2	11
Overexpressed >2.5 ×	-	0	2	0	1	4	1	0

Embryoid body genes (*n *= 196)	168	162	158	154	152	134	156	149
Underexpressed <0.4 ×	-	2	10	6	6	5	6	11
Overexpressed >2.5 ×	-	6	6	0	0	0	3	1
